# The Role of Communities of Residence on Older Mexican Adults’ Likelihood of High Depressive Symptoms

**DOI:** 10.1093/geroni/igaa057.3396

**Published:** 2020-12-16

**Authors:** Paige Downer, Rebeca Wong

**Affiliations:** The University of Texas Medical Branch at Galveston, Galveston, Texas, United States

## Abstract

Exposure to population- and individual-level poverty, poor health, and negative life-events contributes to overall life adversity that may increase older adults risk of depression. We hypothesize that increased neighborhood-level multidimensional poverty and decreased proportions of within-neighborhood health promoting socio-demographic characteristics will be positively associated with depression among older Mexican adults. This study uses data from Wave 1 (2001) and Wave 3 (2012) of the Mexican Health and Aging Study (MHAS). Wave 1 will be used for information for socio-demographic characteristics, including gender, female-headed households, rural settings, and employment status. Wave 3 will be used for information on self-reported depressive symptoms. Information for neighborhood characteristics will come from the 2000 Mexican Census that has been linked with the 2001 MHAS wave. Older Mexican adult’s exposure to multidimensional poverty at the locality (city/town) level will be measured by the proportion of the population aged 15 and older with low education; the proportion of the population with low access to health care services; the percentage of homes with inferior construction materials; and the proportion of the population without sewage and running water. A multivariable logistic regression will be used to model the association between older Mexican adult’s neighborhood and community characteristics in 2001 on depression in 2012. The expected findings will inform government policies to increase access to affordable housing, the availability of health care services, educational and employment opportunities, and public infrastructures such as transportation, water, and sanitation services, and energy services to reduce mental health burden.

